# Human Papillomavirus Vaccine Perceptions Among Noncollege Young Adults and TikTok Influencers: Qualitative Study

**DOI:** 10.2196/80783

**Published:** 2026-02-06

**Authors:** Amelia Burke-Garcia, Dasha Afanaseva, Erin Cutroneo, Kayla Madden, Angela Sustaita-Ruiz, Estefany Rivera Sanchez, Amy Leader

**Affiliations:** 1 Public Health Department National Opinion Research Center Washington, DC United States; 2 Public Health Department National Opinion Research Center Westminster, CO United States; 3 University of Glasgow Glasgow United Kingdom; 4 Department of Medical Oncology Thomas Jefferson University Philadelphia, PA United States; 5 Brilla Media Ventures Miami, FL United States; 6 University of North Carolina at Chapel Hill Chapel Hill, NC United States

**Keywords:** human papillomavirus, HPV, immunization, vaccine hesitancy, TikTok, young adults, non-college, social media influencers

## Abstract

**Background:**

Human papillomavirus (HPV) vaccination is a proven and effective tool for preventing several types of cancers, yet vaccination rates among young adults remain suboptimal, particularly among those not enrolled in 4-year colleges. This population can be more difficult to reach due to fewer established institutional touchpoints, limited engagement with campus-based health services, and greater variability in access to preventive care. At the same time, social media has become a dominant source of information for young adults, with TikTok (ByteDance) emerging as one of the most widely used platforms. Approximately 41% of TikTok’s users are between the ages of 16 and 24 years, making it a potentially important channel for public health communication. However, little is known about how noncollege young adults perceive HPV-related content on TikTok, or how influencers themselves view their role in communicating about vaccination.

**Objective:**

This study explored the perspectives of young adults and TikTok influencers regarding the dissemination and reception of HPV vaccine information on TikTok. The goal was to assess the potential of leveraging influencers as trusted messengers for this hard-to-reach population.

**Methods:**

Researchers conducted 5 focus groups with noncollege young adults, stratified by gender and vaccination status. Each group included 5-8 participants, resulting in a total of 34 individuals. Participants who reported being extremely hesitant about the HPV vaccine were excluded to focus on those more receptive to information. In parallel, researchers recruited 9 TikTok influencers who reached audiences aged 18-25 years and conducted in-depth individual interviews. Influencers represented a diverse mix of identities, follower counts, and content genres, providing varied perspectives on engagement with health-related topics.

**Results:**

Across the focus groups, young adults described regularly encountering or actively seeking health-related information online, with TikTok emerging as a primary or supplementary source for some. However, very few participants reported seeing content specifically related to HPV vaccination. Despite this gap, most expressed openness to such content if it was delivered in a relatable, authentic manner and included concise, relevant facts. Influencers echoed the importance of authenticity, emphasizing that their credibility is grounded in genuine connections with their audiences. Many described frequent, meaningful exchanges with followers about sensitive issues, suggesting comfort in addressing health topics. Influencers noted that they would be willing to share HPV-related content under certain conditions, including alignment with existing content, personal relevance, or participation in a structured campaign or partnership.

**Conclusions:**

Findings suggest that TikTok may be a promising platform to engage noncollege young adults in HPV vaccination messaging. The strong parasocial relationships influencers maintain with their audiences could position them as effective messengers on sensitive health topics. Strategic collaborations with influencers, coupled with carefully crafted, authentic content, may help bridge communication gaps and support increased awareness of HPV vaccination in this underserved population.

## Introduction

### Background

In 2019, the World Health Organization identified vaccine resistance and hesitancy as a top threat to global health [1]. Vaccine hesitancy, defined as patient-level reluctance to receive vaccines, can be conceived on a spectrum that spans from cautious acceptors to outright deniers [2]. Hesitancy is often fueled by concerns about vaccine safety and efficacy, which can be undermined by widespread circulating disinformation and misinformation [3]. Furthermore, vaccine hesitancy is rooted in medical mistrust, which is higher among historically marginalized communities due to generations of systemic and institutional racism, health inequities, and misrepresentation in research [4].

The historic public health crisis resulting from the SARS-CoV-2 (COVID-19) pandemic resulted in significant morbidity and mortality in the United States and around the world, disproportionately impacting racial and ethnic minority communities [5,6]. Similarly, it caused disruptions to many essential health services, including the provision of routine immunizations [7]. Recent data reveal how the human papillomavirus (HPV) vaccination can prevent cervical cancer by 97% [8], but such a disruption to routine HPV vaccination could lead to a reversal of this trend [9,10] and, without an aggressive effort to get vaccination back on track, a rise in cervical and other cancers. Recent studies have shown that there was a decrease in coverage of HPV vaccination programs during the COVID-19 pandemic [10].

Moreover, young adults aged 18-26 years have been less likely to complete the series [11]. A recent population-based study of 18- to 26-year-olds found that men, those with a high-school diploma or less education, and those born outside the United States were less likely to initiate and complete the HPV vaccine series [12]. Now, with the delays in vaccination that have resulted from the COVID-19 pandemic, this group has less time to obtain catch-up vaccinations, which are encouraged up to an age of 45 years, but are most effective when administered at a younger age [13].

Despite this, most HPV vaccination research focuses on adolescents, and the research that has been conducted with young adults has focused on college students and taken place in college and university settings [14-16]. This is partly due to the concentration of this age cohort on college or university campuses and easier access through these networks and systems, the ease of access to the vaccine through university health services, and the fact that most colleges require students to have health insurance to be on campus. Despite having health insurance and proximal easy access to the vaccine, only 41% of 18- to 24-year-olds were enrolled in college in 2019 [17], which means that a majority of this age group—which also needs to receive HPV vaccine messages—is not reachable through these channels. Thus, less is known about 18- to 26-year-olds, particularly those not enrolled in higher education.

Complicating matters is the fact that this cohort may also be less receptive to such messages, and early evidence indicates that decision-making roles about vaccination may be shifting as they increasingly go online for health information and make their own health decisions [18-20], with young adults more likely than adolescents to seek health information online and to make their own health decisions, rather than parents [21,22].

Growth in social media platforms and channels has dramatically changed the nature of communication. There are currently 4.95 billion social media users globally [23], and upwards of 90% of Americans use social media to connect with one another, engage with news content, and share information [24]. Today, there is near-equal participation on social media by age, race, gender, education, and income levels [25], which ensures a wide range and diversity of experiences, opinions, and discourse. Content posted on social media may not undergo scientific vetting, therefore representing a more complex mixture of evidence and personal opinion that can result in misinformation [26,27].

TikTok (ByteDance) became the most downloaded social media app in the country shortly after its official launch in the United States in 2018 and is considered one of the fastest-growing social media platforms, seeing its largest jump in users between 2021 and 2023 [28]. TikTok currently has 1.5 billion monthly active users and was considered one of the fastest-growing platforms in 2025 [29]. Moreover, it is most popular among users younger than 25 years, with 41% of its userbase aged 16-24 years [30]. Many of these users gravitate to the app for its storytelling format, most of which feels relatable and organic versus its more polished and professional counterparts, such as Instagram (Meta). Furthermore, users have been turning to TikTok for more than just entertainment. At least 2 in 5 Americans, especially Gen Z populations, use TikTok as a search engine [31] to look up informative, entertaining, and easily digestible content [32].

An aspect of social media that has emerged in recent years is “online influencers,” which are “everyday people who are incredibly influential within their online social networks” [33]. While millions of people post content to social media, online influencers have established online profiles, talk about a topic or set of topics they are familiar with, and have a cohort of followers who trust their thoughts, opinions, and perspectives. They often garner large followings on social media, which increases the likelihood of engagement with their posts, which, in turn, amplifies the visibility of their content [34].

TikTok’s ability to entertain and build community also paved the way for a new kind of user, one that had the ability to amass large followings and create connections with audiences. Particularly during the COVID-19 pandemic, when people craved connections with others and glimpses into the daily lives of those willing to share them, there was a substantial growth of TikTok influencers [35]. With over 100,000 influencers creating content on the app, its potential impact is hard to ignore [32]. While the growth of influencers on the TikTok platform is lagging behind its competitors, such as Instagram, with its 64 million influencers, there are key aspects of the TikTok platform that may make these influencers more appealing to young users. First, TikTok influencers share content that feels more organic and homemade, likely due to the higher odds of going viral on TikTok, which opens up the opportunity for the everyday user to grow their following and become an influencer [36]. By comparison, Instagram, which emerged nearly a decade before TikTok, is a bit more polished and refined. When considering the importance of connecting with audiences in a way that feels natural and authentic, TikTok appears to have the advantage despite having an overall smaller number of influencers.

For years, social media influencers have been recruited to disseminate information and encourage consumer behaviors [37-39], as they are viewed as highly credible and trustworthy messengers [40]. More recently, health communication and behavioral scientists have begun to shift their attention to partnering with online influencers to spread credible health messages [41-43].

The rise of social media has democratized the ability for many voices, including lay consumers, to share their knowledge about and experiences with vaccination, in both positively and negatively framed messages. The nature of the medium to enable the unfettered dissemination of any kind of content means that both accurate and inaccurate information can easily and quickly be disseminated. In the context of COVID-19, for example, previous studies have found social media to be a major channel contributing to the spread of misinformation, which is often tied to vaccine hesitancy [44].

In fact, much of the misinformation across social media has been vaccine-related. To date, a variety of studies have shown that antivaccine messages receive more attention across social media platforms than provaccine messages [45-49]. On Twitter (subsequently rebranded as X), studies have found a higher percentage of antivaccine messages compared with provaccine messages, and users on YouTube (Google) were also more likely to encounter negative messaging around vaccines [44]. Furthermore, social media enables users to share content with others and contribute to its virality, regardless of whether that content is accurate or originates from a reputable source.

As it relates to TikTok, researchers have found that the algorithm has favored vaccine conspiracy theories, misinformation, and false narratives around the COVID-19 vaccine, likely due to the highly sensationalized and shocking nature of that content, which generates high engagement and interaction from users [50]. Research also shows that social media may impact vaccine knowledge, awareness, and attitudes among people who read this information and may be influential in vaccine uptake [51]. Influencers themselves may either serve to combat misinformation or contribute to its spread. For example, wellness influencers urged their followers to steer clear of the COVID-19 vaccine, stating that homeopathic methods were more effective and safe [52]. That said, influencers who are equipped with evidence-based information and encourage their followers to get vaccinated have been proven to increase vaccination rates [53]. As the field of social media research continues to grow, greater insight into the impact of these messages and messengers on behavior must be better understood.

### Objective

It is within this context that the National Opinion Research Center (NORC), also known as NORC at the University of Chicago, and Thomas Jefferson University (referred to as “the study team” throughout this paper) collaborated to design this study. Guided by the theory of planned behavior (TPB), which has been used to understand and predict HPV vaccination intentions and uptake of young adults enrolled in higher education [54,55], this study sought to better understand the knowledge, attitudes, and beliefs about HPV vaccination as well as TikTok use among 18- to 26-year-old noncollege young adults. As well, the study aimed to understand TikTok influencers’ creative process, relationship with their followers, and their perceptions of the vaccine, and willingness to share HPV vaccination information via TikTok. This paper describes the methods and results of this formative study, as well as implications for policy and practice.

### Theoretical Foundation

This study is guided by the TPB, which posits that behavioral intentions—and ultimately behaviors—are shaped by individuals’ attitudes toward the behavior, perceived social norms, and perceived behavioral control [56]. Applied to HPV vaccination, young adults’ intentions to initiate or complete the vaccine series are influenced by their beliefs about the vaccine’s benefits and risks (attitudes), their perceptions of what peers or trusted figures think they should do (subjective norms), and their sense of ease or difficulty in obtaining the vaccine (perceived behavioral control). Social media platforms like TikTok may shape these determinants by exposing users to normative cues and persuasive messages from influencers, who often serve as relatable and trusted sources for young adult audiences. By examining the knowledge, attitudes, and beliefs of noncollege young adults, as well as influencers’ perceptions and willingness to share HPV-related content, this study provides insights into how the components of TPB may operate within digital spaces to influence vaccination behaviors.

### Research Questions

This study identified four key research questions (RQ):

- RQ1: How do 18- to 26-year-old noncollege young adults think about the HPV vaccine?
- RQ2: How do 18- to 26-year-old noncollege young adults use TikTok?
- RQ3: How do TikTok influencers engage with their followers on TikTok?
- RQ4: What are TikTok influencers’ perspectives about sharing HPV vaccination messages on TikTok?

## Methods

The study team conducted this study from January 2023 to May 2023, collecting qualitative data from two main sources: (1) interviews with select TikTok influencers, and (2) focus groups with noncollege young adults aged 18-26 years.

### Ethical Considerations

Both Thomas Jefferson University and NORC’s Institutional Review Boards reviewed and approved the study’s procedures and protocols (23-01-1132 and 22-03-701, respectively). All participants provided informed consent before participation in the study. To protect privacy and confidentiality, no identifying information was included in the study data and no identifying information is included in this paper or supplementary material. All data were stored securely, accessible only to the research team, and are reported in aggregate to ensure anonymity of participants. As noted below, incentive payments ranging between US $1200 and US $2000 each were provided to influencers via electronic funds deposit, and incentive payments in the form of US $100 e-gift cards were provided to focus group participants.

### Study Administration and Implementation

#### Study Team

The study team consisted of public health communication researchers and qualitative analysts with previous experience in conducting vaccine-related studies and working with online influencers. Several team members had backgrounds in health communication, social media research, and qualitative methods, which informed study design and interpretation. We acknowledge that our professional expertise in vaccine promotion may have shaped the study focus and data interpretation. To minimize bias, we used standardized interview and focus group guides and engaged multiple coders to ensure diverse perspectives were represented during analysis.

#### Instrument and Material Development

The study team created a package of materials for the study, which included (1) influencer recruitment email (Multimedia Appendix 1), (2) screening questions for influencer recruitment (Multimedia Appendix 2), (3) verbal consent statement for telephone interview (Multimedia Appendix 3), (4) influencer interview guide (Multimedia Appendix 4), (5) noncollege young adult focus group screener (Multimedia Appendix 5), and (6) noncollege young adult focus group guide (Multimedia Appendix 6).

All data collection occurred virtually between January and May 2023. The online setting was chosen to facilitate participation across diverse US regions and to mirror the digital environments where participants regularly encounter health content. The following sections describe the methods used for collecting data from 2 distinct sources: TikTok influencers and noncollege young adults.

### Influencer Interviews

#### Study Participant Screening Criteria

The study team sought to include culturally diverse TikTok influencers who reported having an audience that includes or is largely made up of young adults aged 18-26 years, who have completed no more than 1 year of higher education, and who are based in the United States. Given that the purpose of the study was to explore areas of opportunity to promote the HPV vaccine on TikTok, engaging with individuals who have very strong views against the vaccine would not have been productive. Those who responded “strongly disagree” to the following question were excluded: “Rate your level of agreement with this statement: Getting vaccines is a good and safe way to protect you and others from disease.” Given the nature of one of our populations of study, for example, social media influencers, the team identified additional criteria for inclusion to ensure that the influencers were actively participating online by posting content regularly and engaging with their online communities. These were (1) influencers had to have at least a 6% engagement rate (ER) and a minimum of 100,000 followers, and (2) they had to have posted once within the last 3 months.

For this study, ER was defined as the sum of likes, comments, and shares on TikTok posts divided by the number of followers at the time of the campaign, expressed as a percentage. This metric is standard in influencer marketing research and practice, where ER is often used as a proxy for audience connection and authenticity. We applied the ≥6% threshold because influencer marketing benchmarks suggest that TikTok influencers with ERs above 5%-6% are considered to have “high engagement” compared with industry averages of 3%-5% for creators with ≥100,000 followers [57,58]. Using a ≥6% cutoff ensured that influencers selected for this study were highly engaged with their audiences, an important criterion given the exploratory nature of testing receptivity to health communication messages. Follower counts and engagement rates were provided by Brilla Media.

#### Study Participant Recruitment

For this study, 9 influencers were recruited to be participants in this study. We implemented purposive sampling to ensure representation across demographics, and recruitment continued until sufficient variation in perspectives was observed across groups and no new major themes emerged, suggesting thematic saturation. The study team worked with Brilla Media, a multicultural influencer marketing agency, to identify these. To do this, Brilla Media initially sent an alert to their network of over 100 multicultural influencers about the opportunity to participate in a new social media program (Multimedia Appendix 1); this contained general information about the project. In addition to this initial call, Brilla Media also reached out to influencers with whom they had existing relationships, worked before on similar campaigns, or found by searching their network, and reviewing their online profiles for relevance to the project criteria.

#### Participant Screening and Confirmation

Brilla Media compiled a list of all influencers that met the study criteria for review. This included links to social platforms, followers or reach, and any other notes or insights from Brilla Media. The study team then reviewed and approved the influencers who would formally be invited to participate. Influencers were prioritized if their content featured health topics, news, advice, lifestyle, or their personal lives, and therefore, a potential fit for sharing HPV information in a future public health campaign. Upon approval, Brilla Media sent a follow-up email with additional details (Multimedia Appendix 5) about the project, including timing and compensation, to confirm influencer participation in the project. Influencers who were interested signed up using a screening form (Multimedia Appendix 2) to express interest.

#### Influencer Interviews

Each influencer participated in individual virtual interviews, which lasted between 25 and 45 minutes. One research staff member conducted each interview one-on-one using the Zoom platform (Zoom Communications). Per request, 2 interviewees had their agent sit in on the call, but they did not participate in the interview. Interviews were recorded after the influencer gave consent (Multimedia Appendix 3). The research staff members all used the same interview guide to conduct the interview (Multimedia Appendix 4). Interview recordings were securely sent to the Ubiqus transcription service for transcription. As Brilla Media negotiated rates with each interviewee individually and confidentially, after the research team confirmed that an influencer had participated in their interview, Brilla Media issued the previously agreed payment amount (ranging between US $1200 and US $2000, each) to them via electronic funds deposit.

### 18- to 26-Year-Old Noncollege Young Adult Focus Groups

#### Study Participant Screening Criteria

Participants in the focus groups were included if they met the following criteria: (1) aged 18-26 years, and (2) not enrolled in a 4-year university degree program, or had not attained a 4-year university degree.

#### Study Participant Recruitment

The research team partnered with a third-party market research firm, PRC Global Market Research, to recruit participants for the virtual focus groups. PRC recruited these participants through their nationwide database of respondents, reaching out to potential participants with a short description of the project and time commitment needed via email and/or phone to determine their eligibility if interested. Up to 10 participants were recruited for each group, with a goal to seat 8, which allowed for 2 alternates in case of no shows.

#### Participant Screening and Confirmation

PRC recruited a total of 34 participants using the focus group screener (Multimedia Appendix 5) to determine eligibility. Similar to the influencers, individuals who responded “strongly disagree” to the following question were excluded: “Rate your level of agreement with this statement: Getting vaccines is a good and safe way to protect you and others from disease.”

#### Focus Groups

PRC conducted tech checks with participants ahead of the groups, which were held on Zoom, and joined at the start of each group to troubleshoot any tech issues and take attendance. Each group had between 5 and 8 participants and was organized by gender and/or vaccination status, both of which were self-reported by the participants. Furthermore, 2 member teams of the research team (EC, KM, and ERS) administered each group: a notetaker and the interviewer, who used the focus group discussion guide (Multimedia Appendix 6). A subject matter expert with experience in conducting qualitative research with members of the LGBTQ+ (lesbian, gay, bisexual, transgender, queer/questioning, plus [others]) community, who was not part of the main project team, conducted the focus group with transgender and nonbinary participants. Following each focus group, PRC issued the incentive payment of US $100 to each participant via e-gift card. Focus group recordings were securely sent to the Ubiqus transcription service for transcription.

### Analytical Process

#### Codebook Development

After the interviews were completed, the same team that conducted the interviews collaborated on creating two codebooks: (1) interview analysis codebook, and (2) focus group analysis codebook.

These were developed both inductively and deductively—drawing from the literature as well as based on key themes that naturally emerged from the influencer interviews and posts. Previous research on vaccine discourse and message framing across social media platforms, as well as studies on how online influencers disseminate health information and shape audience perceptions [37,43], provided a basis for identifying relevant inductive and deductive codes. Drawing on this literature, the team identified initial themes, such as trust in messengers, credibility of online sources, and peer influence, which were subsequently refined and expanded through an inductive review of the transcripts.

The full project team contributed to codebook development. The team went through several rounds of review of the codebooks to ensure that the team aligned on the codes and scope of the codebooks, therefore ensuring that they were appropriate tools for analysis. Additionally, a sample of content (Interview 1 and Focus T&NB-V Group) was analyzed by 2 independent coders and then compared for analysis. The study team met to review coding discrepancies, resolve these differences through consensus, and make final adjustments to the codebooks. κ scores ranged from 0.42 to 0.86.

#### Coding and Cleaning Data

All focus groups and interviews were then imported into NVivo (version 1.6.1; Lumivero), and the study team used the 2 final codebooks to code the remaining interview and focus group transcripts, with 1 coder assigned to each document. Furthermore, 4 team members (DA, EC, KM, and ERS) who had conducted interviews and focus groups also coded the transcripts. After coding was complete, the coded files were merged among reviewers and exported. In addition, 1 member (EC) of the team then cleaned these exported files to remove duplicate codes.

#### Thematic Analysis of Coded Data

After coded files had been cleaned, the team reviewed the files separately, drawing out key themes that each saw emerging in the data. They then met for an inductive brainstorming session to solidify the key themes and learnings that emerged from the coded files. Through this process, the final primary themes were identified in each of the coded files (interviews and posts) as well as across both of them; they were confirmed through team consensus.

## Results

### Demographic Characteristics of Study Participants

A total of 9 influencers participated in interviews, and 34 young adults participated in focus groups. The demographic characteristics of these influencers and young adults, including gender, race, ethnicity, education, and income, are presented in Table 1.

**Table 1:** Demographic characteristics of study participants.

Characteristic		Influencers (N=9), n (%)	Young adults (N=34), n (%)
**Gender**			
	Cis-female	5 (56)	15 (44)
	Cis-male	2 (22)	14 (41)
	Transgender	1 (11)	2 (6)
	Nonbinary	0 (0)	3 (9)
	Two Spirit	1 (11)	0 (0)
**Race**			
	Caucasian or White	3 (33)	11 (32)
	African American or Black	2 (22)	9 (26)
	Indigenous	2 (22)	0 (0)
	Multiracial	1 (11)	6 (18)
	Hispanic or Latino	1 (11)	4 (12)
	Asian	0 (0)	4 (12)
**Ethnicity**			
	Non-Hispanic	4 (44)	28 (82)
	Hispanic	3 (33)	6 (18)
	Other: Indigenous	2 (22)	0 (0)
**Marital status**			
	Married or common law married	4 (44)	6 (18)
	Single	5 (56)	28 (82)
**Education level**			
	Less than high-school diploma	1 (11)	0 (0)
	High-school diploma	1 (11)	12 (35)
	Associate degree or some college	2 (22)	22 (65)
	College degree	4 (44)	0 (0)
	Graduate school or graduate degree	1 (11)	0 (0)
**Income (US $)**			
	Less than 20,000	1 (11)	2 (6)
	20,000 to <30,000	0 (0)	7 (21)
	30,000 to <40,000	0 (0)	9 (26)
	40,000 to <50,000	1 (11)	6 (18)
	50,000 to <75,000	1 (11)	5 (15)
	75,000 to <100,000	2 (22)	4 (12)
	100,000 or more	1 (33)	2 (6)
	Prefer not to say	1 (11)	0 (0)
**Geographic residence**			
	Rural	0 (0)	—^a^
	Suburban	2 (22)	—
	Urban	7 (78)	—
**Served in US armed forces, reserves, or national guard**			
	No	—	31 (91)
	Yes, but I am not on active service	—	2 (6)
	Yes, I am active service	—	1 (3)
**HPV^b^ vaccination status**			
	Vaccinated, 2 shots	—^c^	14 (41)
	Vaccinated, 1 shot	—^c^	3 (9)
	Not vaccinated	—^c^	17 (50)

^a^Not applicable.

^b^HPV: human papillomavirus.

^c^A diverse sample of influencers were recruited to ensure a mix of perspectives about HPV vaccination. While we excluded those who reported being strongly antivaccine in order to assess opportunities for HPV vaccination communication, participants were not required to disclose their own HPV vaccination status.

### Demographic Characteristics of Influencers

A diverse range of 9 total influencers participated in interviews. Of the total, 5 interviewees were cis-female, 2 were cis-male, 1 was transgender male, and 1 was Two Spirit. Their ages ranged from 21 to 33 years, with the median age being 28 (IQR 23-30) years. Furthermore, 3 interviewees identified as White, 2 as African American or Black, 2 as Indigenous, 1 as Multiracial, and 1 as Mexican. Moreover, 3 of the interviewees identified as Hispanic, Latino, and Latina. Influencers had a diverse range of incomes, with the highest being US $380,000 and the lowest being less than US $20,000 annually. Similarly, there was a wide range of influencers’ highest level of education, with 1 having less than a high-school diploma, 1 with a high-school diploma, 1 with some college, 1 with an associate’s degree, 3 with bachelor’s degrees, 1 with a bachelor’s degree and some graduate school, and 1 with a postgraduate degree. In addition, 4 interviewees were married or common law married, and 5 were single. Of the total, 7 influencers reported residing in urban areas, while 2 reported residing in suburban areas (Table 1).

### Demographic Characteristics of Young Adults

A diverse range of 34 participants took part in 5 focus groups. The groups were stratified according to gender and/or vaccination status:

1. Female, vaccinated group
2. Male, vaccinated group
3. Female, unvaccinated group
4. Male, unvaccinated group
5. Transgender and nonbinary, vaccinated group (T&NB-V; while not required to be vaccinated to participate, all who ended up in the group happened to be vaccinated)

The median age of participants was 22. In total, 32% (11/34) of participants identified as White, 26% (9/34) as Black or African American, 18% (6/34) as Multiracial, 12% (4/34) as Hispanic or Latino, and 12% (4/34) as Asian. Furthermore, 47% (16/34) of participants had an annual household income of US $20,000-US $60,000. Moreover, 65% (22/34) had an associate’s degree, trade school, or some college, while 35% (12/34) had completed high school or a GED. In addition, 82% (28/34) of participants had never been married, while 18% (6/34) were married (Table 1).

### Perspectives on the HPV Vaccine Among Noncollege Young Adults

All groups had some general awareness of HPV being a sexually transmitted infection that can lead to cervical cancer, which is somewhat common, but they reported not hearing a lot of information or news about it. There also appeared to be some confusion about how the disease affects females and males, as well as recommended age groups for vaccination. Generally, people did not feel that they had a lot of knowledge about HPV beyond these basic things.

Related to the HPV vaccine, they generally had heard about it, knew that one existed, and that it helps prevent certain cancers or infections that cause cancers:

Is it to help prevent any type of certain cancers, like, in the future? That’s, like, so far what I know.

They also knew that children should get it, but tended to think that it was for children aged 9 and 10 years. Recommended vaccination for other ages was less well known. As one stated,

I know that I think you get it around age, basically, as a kid, maybe ten or nine, something like that, if I can remember.

Both unvaccinated groups felt like they lacked information about the vaccine and needed to do more research, while the vaccinated groups all reported not needing more information. One participant from the male (unvaccinated group) said,

I just remember that it’s another vaccine to help prevent, but I was kind of confused exactly what it’s exactly preventing, so, that’s something that I’m trying to figure out. I didn’t know if it was more of just, like, a sexually transmitted vaccine that’s preventing everything so you don’t have to use protection, or exactly how it works.

In terms of the safety of the vaccine, some participants thought that the vaccine was safe, but that came more from the vaccine having been around for a while:

It’s been out there a long time. So I took it when I was younger. So it had to have been out there a long time. I’m pretty sure it’s trusted and tested.

Notably, most appeared to have heard of side effects and injuries caused by vaccines, including the HPV vaccine:

The HPV one and Covid one, like [participant] was saying, in particular, I have heard of people getting reactions to it. Like, with the HPV vaccine in particular, I’ve heard people not feeling well or passing out after it. But again, that’s just people on Facebook I hear talking about it, so I don’t know if that’s actually a thing, or if people are just trying to avoid other people getting it. I do think the polio, TDAP, those, like, the childhood ones are what I usually generally always consider safe, like, I’ve gotten those for my kids. But HPV, Covid, and flu I’m just kind of on the fence about.

Many participants who were vaccinated obtained their vaccines as children because of their parents’ decision or a recommendation from their doctor, while others mentioned school requirements as a primary motivator for getting the vaccine. As one participant stated,

I got it for school, and everyone was recommending to take it, along with a bunch of other vaccines.

### Noncollege Young Adults’ Use of TikTok for Health Information

Participants tended to all agree that they heard about information more generally, and health information specifically, on social media first:

I think I for sure hear about a lot of major things from social media first, just because social media has a tendency to spread things like wildfire.

Almost all of them reported that checking sources was important to them, because there was a general understanding that not everything in social media could be believed:

It’s kind of like the good and the bad of social media, it can be really garbage and it can be really good, and how trustworthy it is is kind of up to how much effort you’re putting into using it responsibly.

Checking sources did not necessarily translate to looking for academic or scientific sources to verify the information, though. Rather, they reported that often they are looking for verification from others:

I use comments or refer to comments to sort of verify whether or not the information in the video is correct. Because if people, if the general population thinks that what someone is saying is incorrect, then they’re obviously gonna comment on that, to turn it down. So I usually look at it just to verify if information is right or not… Yeah, yeah. I’m seeing how many people like that comment, which is why they’re probably on the top in the first place. I don’t really click on their profile, I just see the interactions within the comments. So people always respond to the top comments, and I sort of read that whole conversation and see what people say on either side.

And this leads to a lot of codiagnosing, of the act of trying to diagnose someone’s symptoms together:

And then I’ll notice it on, like, people posted forums, they’ll compare each other’s symptoms and kind of find out what it could be, together. And from there, it could lead me to a credible article that one of them posted, like, as a credible source that they’re saying, “Oh, it could be this. Take a look at this. I have these same symptoms.”

Moreover, they noted learning a lot by reading other people’s comments, and they also remarked that reading the comments helped to validate their thoughts or reactions to the online information they were seeing:

And then, looking at the comments, I feel like, is also another big thing for me, like, on videos like that, I then look at the comments. ‘Cause a lot of other people then will share, in the comments, [crosstalk] counter experiences or kind of reaffirm that person’s experience, which I feel like can be helpful to see how reliable it actually is, if you have a lot of people agreeing or not... I’m not looking for a specific thing, I just feel like some people are, like, “Oh, yeah, I had the same experience,” or, “Oh, my daughter, my friend, or whoever, had these same things.” But also, then there are sometimes people who are, like, “Oh, this was not the case for me, at all. I had this and I had a totally different experience.” So, just keep in mind not everyone has the same experience. There’s always people who kind of like have that in the comments, as well, which is good.

As it relates to TikTok specifically, participants tended to think of TikTok as “human,” “transparent,” and “democratic”:

So I think it’s always good to get a human understanding of what’s going on, even if you approach it with hesitation, or you run that and then you verify it again with a more educated source, I think social media is great for looking at what’s actually happening in people’s lives regarding these medical things. Such as, you know, like I said, on the one half of how the medical industry can poorly treat people who have maybe more invisible issues like chronic pain, or how it could clear up my skepticism of Covid by showing me a hospital filled with Covid patients, you know? So I think social media can be really good, just seeing how people in real-life are dealing with the consequences of these things.

Because of this, they perceived the platform as a trusted source that could be used to verify information that was being shared through more mainstream channels:

Yeah, it’s not the fact that TikTok is super reliable, but I think it’s the ease of searching up something and getting videos that answer your questions, you know? So, that’s why I like to use TikTok as a social media platform is because, whenever I have a question, I could just type it in, and then the results that populate are usually informative of that question. But for other social media apps like Instagram, I can’t really type in a question. So, I don’t really get that same search response, which is why I like using TikTok specifically.

Participants also commented on whether they saw HPV and HPV vaccination information–related information on TikTok. Most of them reported not seeing much or not being able to recall seeing anything. A few noted some content that they did remember seeing:

I actually have seen one or two TikToks in, like, the past year, about them, which is why earlier when I said I’ve been thinking about them again, that’s, honestly, why. But then, like, your “for you” page is what TikTok thinks you want to see, right? So, that also dictates a lot of that for me… one of them was about - so, again, in TikTok, you know, people usually talk about these extreme cases. So one was about a girl who had never gotten the vaccine, and it was just, like, she got cancer as a result, and she was just kind of tracking how it led to that and how preventing it could’ve been useful for her. Hers was very much informational, it wasn’t very, like, “Oh, my god, I got cancer and I’m gonna die. Get your HPV vaccine.” It was just, like, “This is kind of what happened to me, like, just keep in mind.” And she also threw in points that her doctor herself told her, too.

Despite this, participants reported that they would be open to seeing this kind of content on TikTok but had recommendations for what they would like to see in terms of that kind of content. Most mentioned wanting natural, unbranded, unscripted content that felt authentic. They notably said that they would want the video made with a phone to give it a more personal quality. They also noted that they wanted to hear personal stories:

I would be more inclined to watch a video that was describing somebody’s personal experience.

Some mentioned that hearing statistics or health care providers talking about the disease and the vaccine would be interesting, but that they *“don’t know if [they would] watch the whole thing, but… [they] would feel like it's good that it’s out there.”* They did note that they would want videos to include *“more background about what this disease is, how people get it, and if there’s a cure or not, what the vaccine does to help prevent HPV.”* For both vaccinated and nonvaccinated, they said that they would like to see this kind of information shared via influencers, people with personal experience, and *“*s*omebody, like, my age who I didn't know, if they sounded confident and enthusiastic.”* Only vaccinated participants said that they would like to see influencers and doctors talking about this topic together.

### Influencer-Follower Engagement on TikTok

TikTok influencers noticeably reported having very close relationships with their followers, perhaps some of the closest of any social media platform. One noted,

I go live a lot, so I get to talk to my followers in real time and thoroughly get to know them, because for me, I don’t believe in I’m here and they’re here. For me, I like to personally get to know my followers, so I can name them by name, I can come into a live and be like, “Oh, I’ve missed you. What have you been up to?” I follow some of my followers back just to get to know them, because I know so many of them are following me on this journey.

They referred to these relationships as “familial,” like an “older sister,” “aunt,” or “uncle,” and reported “knowing their followers by name.” One participant who shared that her followers say that she’s relatable and that they see her as an “older sister” described being vulnerable with her audience, which is why she thinks she has been able to connect with her audience so much:

I would say that my relationship with my followers is that of like being an auntie or an uncle. Somebody who travels a lot, who gets a lot of really unique experiences, who has a lot of life experience, who’s done a lot of different things.

Participants reported that these relationships result in personal and deep conversations with their followers:

I feel like I’m speaking to my friends and my parents, is sort of how I address it. Friends who are not necessarily technical - my parents are not technical. So, that’s how I - I kind of feel like loose friends with my audience, even though I obviously haven't met more than a handful of them.

They also noted that they were often asked for their advice on topics and for their help answering questions:

A lot of my followers will tag me in videos that are really controversial. Videos from members of the community, of the Indigenous community. Even legislation or like some crises that are happening in different communities. They’ll tag me and say, “Hi, [NAME]. Like, what are your thoughts on this?” or “You know, are there any words of like wisdom or advice you can give for people going through this?”

One participant noted how his followers messaged him for advice on being transgender, struggling with their identity, or their environment (meaning, friends and family). He noted that he would try to respond in ways that were supportive and helpful:

He had messaged me years ago about questioning his identity, and I gave him some advice back then. Then he messaged me again like a week ago and he was like, “I finally came out and you’ve helped me.” I like to be able to be somebody that people can look up to and see that it’s okay and it’s normal. I don’t know, like somebody that I wish I had when I was younger to know. I feel like I would know more.

Finally, the participants noted that one of the main ways those one-on-one conversations happen is via personal direct messages on TikTok. As one noted,

I get messages, at least like a dozen messages every single day. I guess we live in this time where people are surprised that I respond back to them and I try to respond back to every single person that messages me with more than just a, “Hey, thank you.” You know ask, if they ask questions try to give responses back and everything. So, I have a pretty close relationship with my followers. I think they feel comfortable with me. I think that’s one of the big things that makes me a good content creator I guess, is that I’m able to be approachable, that people are able to talk to me and I respond back to them with a comment to their messages. So I would say it’s a pretty cool relationship with them.”

Because of these kinds of relationships and conversations, TikTok influencers, in particular, feel that they are helping to build community and can be incredibly persuasive with their followers:

A lot of people just end up commenting and saying, “Oh my God, wow, I’m so happy that you brought this up because I'm dealing with this” and it just becomes a big community.

One participant noted how many of her indigenous followers follow to connect with their heritage, learn, and/or support her as an influencer. She also noted, however, how she had a following of non-Indigenous people, and they seemed to follow her to learn more about her and her culture. The influencers felt that this kind of community-building often leads to conversations that were not traditionally held openly or freely, which present learning opportunities:

I feel like for the non-Indigenous people it’s just to learn… That’s what I get usually in my messages when people are like… “I never really learned a lot about Indigenous culture.” [or] “they don’t teach it so, like, that’s why I follow you.” And for the Indigenous people some of them are reconnecting. So, I would say the reasons [for indigenous folks] are kind of the same as the non-Indigenous folks. But some… Indigenous people just follow me because I’m Indigenous and, you know, just to show their support I would say.

### TikTok Influencers’ Perspectives on Sharing HPV Vaccine Messages

Influencers varied in the level of health information they shared on their platforms. Some had platforms that focused on health subjects, while others said that while they had not yet posted any health-related content, they would consider doing so in the future. One noted,

I don’t think I have ever done it myself just ‘cause it doesn’t fall into like my category of stuff that I do.

When asked whether they had posted about vaccines on their platforms, the influencers were split—about half had, while the other half had not. The respiratory syncytial virus and COVID-19 vaccines were popular topics to post about. The HPV vaccine tended to be talked about less often than other vaccines, but for those who had posted about it, they sought to tailor their content to address certain misconceptions about the vaccine:

There’s a lot of misinformation about what types of injuries may result from HPV vaccination, and the VAERS system. So, it changes a little bit depending on what I think are the public’s main issues with each of the vaccines.

Overall, it appeared that TikTok influencers were not talking about the HPV vaccine but were open to it.

## Discussion

### Findings

This study aimed to better understand the knowledge, attitudes, and beliefs about HPV vaccination, as well as TikTok use, among 18- to 26-year-old noncollege young adults. In addition, it sought to explore TikTok influencers’ creative processes, relationships with their followers, and their perceptions of the HPV vaccine, including their willingness to share vaccine-related information via TikTok. Findings indicate that while few young adults reported seeing HPV vaccine content on the platform, many were receptive to receiving such information if it was presented authentically and included clear, relevant facts. Influencers emphasized the importance of their close, trust-based connections with followers and expressed a willingness to post about HPV vaccination when it aligned with their content or personal experiences. Together, these results suggest that TikTok, and the influencers who engage audiences on the platform, may offer a promising avenue for reaching noncollege young adults with HPV vaccination messaging. The findings highlight the critical roles of authenticity, audience connection, and strategic content integration in designing effective social media interventions for this population.

The TPB links people’s attitudes toward a behavior with their intentions to engage in said behavior [56] and seeks to contextualize those beliefs and behaviors within larger social contexts and take into consideration how people perceive what others (eg, peers and people of importance) think about engaging in the behavior. This study extends previous work related to the TPB by identifying ways in which young adults’ health-related attitudes and decision-making may be influenced by peer-to-peer interactions on TikTok. Notably, across both unvaccinated and vaccinated young adults, participants assessed the credibility of health information they were exposed to—online and offline—and made decisions about how to think about those health topics based on popular comments on TikTok posts and interactions with TikTok influencers, whom they trust.

Moreover, our findings further align with and extend existing literature about social media and vaccine communication. Previous studies have shown that social media can shape vaccine knowledge, awareness, and attitudes, particularly among young adults who seek health information online [51]. Moreover, our finding that influencers’ authenticity and relatability make them trusted messengers complements other studies that have found that partnering with social media influencers increased positive attitudes toward vaccination [37]. Our results also echo previous studies showing that narrative storytelling is an effective approach for addressing HPV vaccine hesitancy, as demonstrated by our findings that TikTok users prefer personal and unscripted stories [59]. Taken together, our results contribute to this growing body of research by highlighting TikTok’s unique combination of participatory culture, peer validation through comments, and influencer-follower intimacy as potential mechanisms for strengthening HPV vaccine communication efforts.

Related to HPV and the HPV vaccine, this study elucidates that there appears to be both a lack of knowledge, especially among the unvaccinated groups, as well as a lack of HPV vaccine content on TikTok. Thus, our findings underscore the critical role that a lack of knowledge may play in attitudes toward the HPV vaccine and decision-making about it. Skepticism can be attributed to a lack of knowledge [60], and many study participants, particularly in the unvaccinated groups, expressed having a lack of information or knowledge on the topic and a need to do more research about it. To address this gap in knowledge, some participants expressed searching for information on TikTok given its accessible, short-form content. Yet, little to no HPV vaccine-related content exists on the platform. Thus, the mismatch between need and availability may be contributing to the lack of knowledge, awareness, and decision-making around the vaccine.

Given this lack of knowledge about HPV among this audience, particularly among those who are nonvaccinated (as well as the lack of such information on this channel), strategies focused on addressing these knowledge and information gaps are critical. Noncollege adults have less access to information about the HPV vaccine as well as reduced access to health care services compared with those in college or university systems. It is therefore worth exploring ways in which TikTok may be leveraged to reach this audience with this kind of information, especially since these individuals commonly access this platform.

Related, the unique aspect of storytelling and personal narratives on TikTok is important to consider when it comes to understanding the role that these influencers may play in sharing such information with their followers and the impact that may have on their followers’ HPV vaccine-related behaviors. It is well-documented in the literature that personal narratives create space for the acceptance of new information and have been well-documented as a communication approach that can effectively lead to higher HPV vaccination rates [59]. Many participants expressed a preference for receiving health information in authentic and unscripted formats and wanted to hear about people’s real experiences with a health condition like HPV and getting the HPV vaccine. Matched with TikTok influencers’ positions as deeply trusted sources and their willingness to share such information, under the appropriate conditions, there is an opportunity and need to further explore this channel and this set of messengers as potentially powerful conveyers of the HPV vaccination information that this age cohort is seeking—and how this might drive changes in HPV vaccine-related behaviors.

Ultimately, these findings offer guidance for communicators in terms of messaging about HPV and the HPV vaccine. Public health communicators should consider how to work with TikTok influencers to share their messages, especially about the HPV vaccine, taking advantage of their position of influencer and empowering them to tailor and personalize HPV vaccine messages as only they can—in authentic and personal ways. Their storytelling approaches, combining narrative and didactic content, can help amplify public health and immunization messages and begin to address knowledge and awareness gaps that remain strikingly persistent—even nearly 20 years after the vaccine was approved in the United States.

### Limitations

This study is not without limitations. First, the study did not compare results with those of college-educated young adults, but rather explored the opinions of a diverse set of young adults who were not enrolled in higher education. It is therefore possible that some confounding variables may exist that could contribute to the differences or similarities between these 2 groups. Second, this study was exploratory in nature and therefore did not explore the effects of HPV vaccination messaging shared by influencers on noncollege young adults that could otherwise be observed through an intervention-based or longitudinal study. Finally, the study had a small sample size to represent a large portion of the population, and therefore, these results are not generalizable.

### Conclusions

Noncollege young adults aged 18-26 years’ experience substantial challenges accessing vital information about HPV and the HPV vaccine. This has resulted in lower vaccination rates and higher rates of related infections and cancers. Novel strategic approaches are needed to directly engage young adults with timely and relevant HPV vaccine information. This study’s findings suggest that the power of narrative storytelling combined with the trusted relationships that exist between an influencer and their followers—as only exists on TikTok—may be helpful and persuasive to this audience.

## Appendix

Multimedia Appendix 1 Influencer recruitment email.

Multimedia Appendix 2 Screening questions for influencer recruitment.

Multimedia Appendix 3 Verbal consent statement for telephone interview.

Multimedia Appendix 4 Influencer interview guide.

Multimedia Appendix 5 Noncollege young adult focus group screener.

Multimedia Appendix 6 Noncollege young adult focus group guide.

## Abbreviations

HPV: human papillomavirus
LGBTQ+: lesbian, gay, bisexual, transgender, queer/questioning, plus (others)
NORC: National Opinion Research Center
RQ: research question
TPB: theory of planned behavior

## Data Availability

The datasets generated or analyzed during this study are available from the corresponding author on reasonable request.
