# Supplementary material for: Human Papillomavirus Vaccine Perceptions Among Noncollege Young Adults and TikTok Influencers: Qualitative Study
Source: JMIR Form Res. 2026 Feb 6;10:e80783. doi: 10.2196/80783 (PMC12924042; doi:10.2196/80783)
Supplement: Multimedia Appendix 1 [file formative_v10i1e80783_app1.docx]

**Appendix 1. Influencer Recruitment Email**

Initial Email:

Dear [insert name of influencer]:

I hope that my email finds you well! [insert any tailoring based on personal history with this influencer]

I am reaching out to you because my company, Brilla Media Ventures, LLC., is working with NORC at the University of Chicago and Thomas Jefferson University to recruit influencers like yourself for a timely research study around HPV (Human papillomavirus). We are eager to hear about your insights on your content production process for TikTok and attitudes toward creating health content on TikTok, your personal health information-seeking habits, and your perspectives on health content on TikTok and the HPV vaccine.

We are looking to conduct **paid interviews** with TikTok influencers who reach young adults ages 18-26 that have completed no more than one year of higher education. Influencers will be asked to participate in one virtual, 45-minute interview where they will be asked about their overall approach to TikTok content creation; their willingness to share health-related information, especially about HPV vaccination; barriers to doing so; and their approach to selecting information sources and messages for health-related content. **Influencers will not be required to create or share any content.**

Participation in this study will include the following:

- Payment to compensate for the influencer's time **in the amount of [insert value depending on the influencer].**
- We can also share with you all results from the work we do once they are available.

Due to the sensitivity of this study, we ask that you please keep all information regarding this project private until we are able to share the results more widely.

We greatly appreciate you considering this opportunity. Your participation would help us grow our understanding of how to encourage HPV vaccination among young adults, especially those who may be getting health information online versus more traditional settings.

If you are interested in participating, please respond to this email clarifying your interest and with responses to the following questions:

1. Does your TikTok account have a minimum of 100,000 followers?
2. Yes
3. No

1. Is at least 25% of your TikTok audience young adults between the ages of 18-26?
2. Yes
3. No
4. Have you ever heard of the HPV vaccine?
5. Yes
6. No

1. *Rate your level of agreement with this statement:* Getting vaccines is a good and safe way to protect you and others from disease.

a.  Strongly agree

b.  Agree

c.  Neither agree nor disagree

d.  Disagree

e. Strongly disagree

Thank you,

[insert name of influencer recruiter]

Follow-Up Email if no Response:

Dear [insert name of influencer]:

I wanted to take a moment to quickly follow up on my previous email. When you have a moment, please let me know if you are interested in finding out more and possibly participating.

Thank you,

[insert name of influencer recruiter]

Follow-Up Email if no Interest from Influencer:

Hi [insert name of influencer]:

Thank you so much for getting back to us. We understand that you do not wish to be involved. Please feel free to let us know if anything changes.

Thank you,

[insert name of influencer recruiter]

Thank You Email for Influencer:

Hi [insert name of influencer]:

Thank you for participating in this important and timely research study around HPV. Your participation helps us to better understand the influencer content production process for TikTok and influencers’ attitudes toward creating health content on TikTok, their personal health information-seeking habits, and their perspectives on health content on TikTok and the HPV vaccine.

Please remember that due to the sensitivity of this study, we ask that you keep all information regarding this project private until we are able to share the results more widely. We are happy to share final study results with you once they are available, at which point you will be welcome to circulate and share with your community.

Thank you,

[insert name of influencer recruiter]
