# Supplementary material for: Human Papillomavirus Vaccine Perceptions Among Noncollege Young Adults and TikTok Influencers: Qualitative Study
Source: JMIR Form Res. 2026 Feb 6;10:e80783. doi: 10.2196/80783 (PMC12924042; doi:10.2196/80783)
Supplement: Multimedia Appendix 2 [file formative_v10i1e80783_app2.docx]

**Appendix 2. Screening Questions for Influencer Recruitment**

**Eligibility for Merck Influencers (Questions 1-4):**

- Minimum of 100,000 followers
- Minimum 6.7% engagement rate
- At least 25% of their audience is young adults ages 18 to 26
- Not anti-vax

1. Does your TikTok account have a minimum of 100,000 followers?
2. Yes
3. No **-> STOP, not eligible.**
4. We intend to record these interviews for our records. They will be sent to a third-party company securely for transcription. We are transcribing interviews to help with analysis. Your responses will remain anonymous during transcription as the transcription service will not have access to your name or any of your information. Your name will not be identified or associated with any specific responses, and it will not appear in any published materials which result from this research. Do you consent to participating in a recorded focus group?
   1. Yes
   2. No **-> STOP, not eligible**
5. Is at least 25% of your TikTok audience young adults between the ages of 18-26?
6. Yes
7. No **-> STOP, not eligible.**
8. Have you ever heard of the HPV vaccine?
   1. Yes
   2. No **-> STOP, not eligible.**
9. *Rate your level of agreement with this statement:* Getting vaccines is a good and safe way to protect you and others from disease.

a. Strongly agree

b. Agree

c. Neither agree nor disagree

d. Disagree

1. Strongly disagree **-> STOP, not eligible.**

FOR THOSE WHO PASS THE SCREENER QUESTIONS:

1. What is your gender?

a. Male

b. Female

c. Non-binary

d. Transgender

e. Other

1. What is your age?
   1. (fill in blank)
2. What is your ethnicity?
   1. Hispanic or Latino/Latina
   2. Not Hispanic or Latino/Latina
   3. Other (please specify)
3. What is your race (choose all that apply)?
   1. American Indian or Alaska Native
   2. Asian
   3. Black or African American
   4. Native Hawaiian or Other Pacific Islander
   5. White
   6. Biracial
   7. Multiracial
   8. Don’t want to say
4. What is your income?

a. Less than $20,000

b. $20,000 - $40,000

c. $40,000 - $60,000

c. $60,000 - $80,000

d. $80,000 - $100,00

e. Greater than $100,000

f. Prefer not to answer

1. What is the highest degree or level of school you have completed?
   1. Less than a high school diploma
   2. A high school diploma/ GED
   3. An associate degree, trade school, or some college
   4. A college degree
   5. A post-college or graduate degree
2. What is your marital status?
   1. Single (never married)
   2. Married, or in a domestic partnership
   3. Widowed
   4. Divorced
   5. Separated
3. Please fill out the following contact information:
4. Name:
5. Primary TikTok handle (URL):
6. Email address:
7. State:
