# Supplementary material for: Human Papillomavirus Vaccine Perceptions Among Noncollege Young Adults and TikTok Influencers: Qualitative Study
Source: JMIR Form Res. 2026 Feb 6;10:e80783. doi: 10.2196/80783 (PMC12924042; doi:10.2196/80783)
Supplement: Multimedia Appendix 3 [file formative_v10i1e80783_app3.docx]

**Appendix 3. Verbal Consent Statement for a Telephone Interview**

Thank you for agreeing to be interviewed. We are eager to hear about your insights on your content production process for TikTok and attitudes toward creating health content on TikTok, your personal health information-seeking habits, and your perspectives on health content on TikTok and the HPV vaccine. Your participation is entirely voluntary. You may stop the interview at any time or decline to answer a specific question.

The risks of partaking in this study are very low. You may be uncomfortable or become anxious by some of the questions. If you find this happening, please let us know and we will immediately pause the interview. In addition to this risk, there may also be risks that are not known at this time.

You may not personally benefit from taking part in this research, but other people may be helped by what is learned.

We want to assure you that any information you provide today will remain strictly confidential.  Your name will not be identified or associated with any specific responses, and it will not appear in any published materials which result from this research.

If you have questions about this research, you can call Dr. Amelia Burke-Garcia at 301-634-5437 or email her at BurkeGarcia-Amelia@norc.org.  Also, if you have questions about your rights as a research participant, you can contact the NORC Institutional Review Board Administrator, toll free, at 866-309-0542.

**Would you like to participate in the study?**

*Yes (Agree)*

*No (Disagree)*

*(Include copies of questionnaire(s) with submission to IRB.)*
