# Supplementary material for: Human Papillomavirus Vaccine Perceptions Among Noncollege Young Adults and TikTok Influencers: Qualitative Study
Source: JMIR Form Res. 2026 Feb 6;10:e80783. doi: 10.2196/80783 (PMC12924042; doi:10.2196/80783)
Supplement: Multimedia Appendix 4 [file formative_v10i1e80783_app4.docx]

**Appendix 4. Influencer Interview Guide**

Thank you for taking the time to speak with me today. My name is {Name~~}~~, and I work for NORC at the University of Chicago/Thomas Jefferson University. On behalf of Merck and in collaboration with NORC at the University of Chicago/Thomas Jefferson University, NORC/TJU is conducting this work to learn how you create content for TikTok, your thoughts on health content on TikTok, and your perspective on the HPV vaccine. Your participation is entirely voluntary. You may stop the interview at any time or decline to answer a specific question. I am the interviewer, and you are the expert – all of your thoughts and responses are appreciated. I will remain neutral about the topic throughout the interview. This interview will take 45 minutes.

In addition, we will send the audio recording of this interview to a third party for transcription. We are transcribing interviews to help with analysis. Your responses will remain anonymous during transcription, as the transcription service will not have access to your name or any of your identifying information.

**Before we get started, I will read you a brief consent statement to ensure that you want to participate in this research. OK?**

Thank you for agreeing to be interviewed. The risks of partaking in this study are very low. If you become uncomfortable or anxious because of any of the questions, please let us know and we will immediately pause the interview. In addition to this risk, there may also be risks that are not known at this time.

You may not personally benefit from taking part in this research, but other people may be helped by what is learned.

We want to assure you that any information you provide today will remain strictly confidential. Your name will not be identified or associated with any specific responses, and it will not appear in any published materials which result from this research.

If you have questions later, you can call the Project Leader, Dr. Amelia Burke Garcia, at 301-634-5437 or email her at [BurkeGarcia-Amelia@norc.org](mailto:BurkeGarcia-Amelia@norc.org). If you have questions about your rights as a project participant, you may call the NORC Institutional Review Board Administrator toll-free at 866-309-0542.

**Would you like to participate in the study?**

*Yes (Agree)*

*No (Disagree)*

Ok, let’s get started.

**Background:**

Let’s start at the beginning. Can you tell me a little bit about how you got started on TikTok? How long have you been posting on TikTok?

How would you describe your content generally? What do you like to post about?

Probe for topics

Now I’d love to know about your followers and your relationship with them. How would you describe your followers? In other words, how would you describe your typical audience?

How would you describe your relationship with your followers?

Dig in on: Why do you feel like they follow you?

Do you ever interact with your followers? How/how often?

Do your followers ask you for recommendations?

**Content Development Discussion**:

I’d now love to hear more about your approach to identifying and developing content specifically for TikTok.

Can you talk me through your process of creating content for TikTok? What does the process look like from when you start to when you post?

Are you on any other platforms?

**If yes to being on other platforms:** What do you like about TikTok versus other platforms?

**If yes to being on other platforms:** Does your TikTok content creation process differ from your content creation for other platforms?

How do you choose your topics?

How would you describe the general tone of your posts (for example, funny, serious, informational, about your personal life, etc.)?

How do you decide how to frame your messages?

As an influencer, what is your goal or purpose in posting on TikTok?

Do you think your TikTok posts change people’s minds or influence people?

How so? Why?

**Attitudes Toward Health Content:**

Thank you for sharing that. I’d now love to hear about your attitudes toward health content on TikTok.

Do you follow health-related accounts on TikTok?

Do you get health content from others on TikTok?

**If yes to following or getting health content from others**: What type of content do you look for? Has it been helpful in any way?

Probe on:

- Specific topics
- What did you like?
- Who did the content come from?
- What about that source did you like?

Have you posted content about health topics on TikTok?

**If yes to posting health content:** Why have you posted about health content? What motivated you?

**If yes to posting health content:** Which health topics?

**If yes to posting health content:** Have you ever posted about vaccines?

**If yes to vaccines**: Which vaccines?

**If yes to vaccines**: What was your message?

**If yes to vaccines**: What was the reaction from your followers?

**If yes to vaccines**: Have you ever posted about the HPV vaccine?

**If yes to HPV vaccine**: What was your message?

**If yes to HPV vaccine**: What was the reaction from your followers?

**If NO** **to health content**: Why do you feel like you haven’t posted health content?

**If NO** **to health content**: Would anything make you interested in posting health content?

**If NO** **to health content**: Would you ever consider posting content about vaccines? Do you think anything could motivate you to do so?

**If NO** **to health content**: Would you ever consider posting content about the HPV vaccine, specifically? Do you think anything could motivate you to do so?

**If NO** **to health content**: What information would you want about the HPV vaccine before posting? Where would you look for the information?

**Health Information-Seeking Discussion:**

Thank you for that information. I’d love to know about your personal health information-seeking habits.

What sources do you go to when you need health information?

What health information sources are most trustworthy to you?

Do you look to other people’s TikTok posts for health information and advice?

If you were to look up information for making a TikTok post on the HPV vaccine, where would you look?

**To be clear, the purpose of this is not to ask you to create a post, but we’d love your expertise and insights into what a potential HPV vaccine post would look like from an influencer like yourself.**

So in a hypothetical where you were to create an HPV vaccine post, what would your message be?

Do you have a sense of what the tone of the post would be?

Do you think you’d be speaking, or would something else be happening?

**To wrap up, I’d like to go over some brief demographic questions:**

How would you describe your gender?

a.  Male

b.  Female

c.  Non-binary

d.  Transgender

e.  Other

What is your age?

1. ______

What is your ethnicity?

- 1. Hispanic or Latino/Latina
  2. Not Hispanic or Latino/Latina
  3. Other (please specify)

What is your race (choose all that apply)?

- 1. American Indian or Alaska Native
  2. Asian
  3. Black or African American
  4. Native Hawaiian or Other Pacific Islander
  5. White
  6. Biracial
  7. Multiracial
  8. Don’t want to say

What is your income?

a Less than $20,000

b.  $20,000 - $40,000

c.  $40,000 - $60,000

c.  $60,000 - $80,000

d.  $80,000 - $100,00

e.  Greater than $100,000

f.  Prefer not to answer

What is the highest degree or level of school you have completed?

1. Less than a high school diploma
2. A high school diploma/ GED
3. An associate degree, trade school, or some college
4. A college degree
5. A post-college or graduate degree

What is your marital status?

1. Single (never married)
2. Married, or in a domestic partnership
3. Widowed
4. Divorced
5. Separated

What state do you live in?

a. ___________________

**Thank you so much for your time! Brilla Media will contact you in the next X days with your compensation for participating in today’s interview.**
