# Supplementary material for: Human Papillomavirus Vaccine Perceptions Among Noncollege Young Adults and TikTok Influencers: Qualitative Study
Source: JMIR Form Res. 2026 Feb 6;10:e80783. doi: 10.2196/80783 (PMC12924042; doi:10.2196/80783)
Supplement: Multimedia Appendix 5 [file formative_v10i1e80783_app5.docx]

**Appendix 5. Non-College Young Adult Focus Group Screener**

**Eligibility for Focus Groups (Questions 1-4):**

- Aged between 18 to 26
- Not enrolled or have not attended higher education
- Regular TikTok user
- No extreme anti-vax attitudes

1. Are you between 18 and 26 years old?
   1. Yes
   2. No **-> STOP, not eligible.**
2. We intend to record these focus groups for our records. They will be sent to a third-party company securely for transcription. We are transcribing interviews to help with analysis. Your responses will remain anonymous during transcription as the transcription service will not have access to your name or any of your information. Your name will not be identified or associated with any specific responses, and it will not appear in any published materials which result from this research. Do you consent to participating in a recorded focus group?
   1. Yes
   2. No **-> STOP, not eligible**
3. What is the highest degree or level of school you have completed?

a. Less than a high school diploma

b. A high school diploma/ GED

c. An associate degree, trade school, or some college

d. A college degree **-> STOP, not eligible.**

e. A post-college or graduate degree **-> STOP, not eligible.**

1. How often do you go on TikTok?
   1. Several times a day
   2. Once a day
   3. Several times a week
   4. Once a week **-> STOP, not eligible.**
   5. Less than once a week **-> STOP, not eligible.**
   6. Never **-> STOP, not eligible.**
2. *Rate your level of agreement with this statement:* Getting vaccines is a good and safe way to protect you and others from disease.
   1. Strongly agree
   2. Agree
   3. Neither agree nor disagree
   4. Disagree
   5. Strongly disagree No -> **STOP, not eligible.**

FOR THOSE WHO PASS THE SCREENER QUESTIONS:

1. Which resources do you trust for health information (multi-selection)?
   1. Doctors or Health Care Providers
   2. Health Officials (CDC, WHO)
   3. News Media
   4. Elected Officials (President, Governor, Mayor)
   5. Social Media Influencers (Not Celebrities)
   6. Celebrities
2. Have you gotten the HPV vaccine?
   1. Yes, I have received both HPV shots and I am fully vaccinated
   2. Yes, I have received one HPV shot but I have not received my second dose yet
   3. No
3. What is your gender identity?
   1. Male
   2. Female
   3. Non-binary
   4. Transgender
   5. Other
4. What is your age?
   1. (fill in blank)
5. What is your ethnicity?
   1. Hispanic or Latino/Latina
   2. Not Hispanic or Latino/Latina
   3. Other (please specify)
6. What is your race (choose all that apply)?
   1. American Indian or Alaska Native
   2. Asian
   3. Black or African American
   4. Native Hawaiian or Other Pacific Islander
   5. White
   6. Biracial
   7. Multiracial
   8. Don’t want to say
7. Have you ever served on active duty in the U.S. Armed Forces, Reserves, or National Guard?
   1. Yes, I am active service
   2. Yes, but I am not on active service now
   3. No
8. What is your household income?

a. Less than $20,000

b. $20,000 - $40,000

c. $40,000 - $60,000

c. $60,000 - $80,000

d. $80,000 - $100,00

e. Greater than $100,000

f. Prefer not to answer

1. What is the highest degree or level of school you have completed?
   1. Less than a high school diploma
   2. A high school diploma/ GED
   3. An associate degree, trade school, or some college
   4. A college degree
   5. A post-college or graduate degree
2. What is your marital status?
   1. Single (never married)
   2. Married, or in a domestic partnership
   3. Widowed
   4. Divorced
   5. Separated
3. Please fill out the following contact information:
4. Name:
5. Email address:
6. State:
