# Supplementary material for: Human Papillomavirus Vaccine Perceptions Among Noncollege Young Adults and TikTok Influencers: Qualitative Study
Source: JMIR Form Res. 2026 Feb 6;10:e80783. doi: 10.2196/80783 (PMC12924042; doi:10.2196/80783)
Supplement: Multimedia Appendix 6 [file formative_v10i1e80783_app6.docx]

**Appendix 6. Non-College Young Adult Focus Group Screener**

**STARTING 15 MINUTES BEFORE START TIME!**

**INTRODUCTION (5 minutes):**

Welcome. Thank you for joining our discussion today. My name is {Name}, and I work for NORC at the University of Chicago/Thomas Jefferson University. On behalf of Merck and in collaboration with NORC at the University of Chicago/Thomas Jefferson University , NORC/TJU is conducting this work to learn about young adults’ HPV vaccine knowledge and social media attitudes. On the session with me is {Name} who will be assisting with taking notes. We also have a representative from the recruitment firm, who is just verifying who is on the line for incentive purposes.

Our discussion will take about 60 minutes. Our goal today is to learn about your knowledge about HPV and the HPV vaccine, your attitudes towards vaccines, your awareness of vaccine access, your health information-seeking habits, and the role of social media in your health decision-making. Please share your open and honest feedback with me to help us best understand your thoughts and feeling related to HPV and the HPV vaccine. There are no wrong answers, and your opinions are incredibly valuable to use.

**INFORMED CONSENT (7 minutes):**

Before we get started, I would like to go over some information about your participation in this focus group. During our discussion, I will ask questions related to the HPV vaccine, your health information-seeking habits, and the role of social media in your health decision-making. I understand that this may be a sensitive topic. Your open and honest opinions are appreciated, but please know that your participation is voluntary, and you may choose to skip any questions or end the discussion at any time. Also, everything shared during this focus group will be confidential, and we ask that you help us with this by keeping information discussed today within this group. We kindly request all of you to keep others responses confidential and also ask that each person speak one at a time so that everyone’s thoughts can be heard.  In addition, we will send the audio recording of this focus group to a third party for transcription. We are transcribing interviews to help with analysis. Your responses will remain anonymous during transcription as the transcription service will not have access to your name or any of your information. Your name will not be identified or associated with any specific responses, and it will not appear in any published materials which result from this research.

If you have questions at a later point, you can call the Project Leader, Dr. Amelia Burke Garcia, at 301-634-5437 or email her at [BurkeGarcia-Amelia@norc.org](mailto:BurkeGarcia-Amelia@norc.org). Also, if you have questions about your rights as a project participant, you may call the NORC Institutional Review Board Administrator, toll free, at 866-309-0542.

NORC would like to record this discussion to ensure our notes are as accurate and comprehensive as possible. We will send the recording to a third party for help transcribing the conversation. The file we send out will not have any identifiable information pertaining to you, and you will not be identified or linked to anything you say today in any way.

Do you have any questions before we begin?

I will now separate each of you into a separate Breakout Room to collect consent to partipcate and record individually.

[In the individual room]

Do you consent to participate in this focus group?

**[All parties must say “yes” to proceed. If “yes” then proceed. If “no” then thank them for their time and remove them from the focus group.]**

**Do you consent to us recording the focus group conversation?**

**[If all parties indicate “yes“, proceed and record the focus group.]**

**[If anyone says no, then notify Haley from Plaza and they will be removed and notified why.].**

**END BREAKOUT ROOMS.**

Thank you all for your time with the consent process. We can now begin the discussion.

**ICE BREAKER (5 minutes):**

First, please tell me your first name only and if you could be any superpower, what would it be. I will start {Start}.

**KNOWLEDGE ABOUT HPV AND HPV VACCINE** **(7 minutes):**

That was fun! I’d love to move us onto the main discussion. To get started,

I’d love to hear from each of you, what do you know about HPV or the human papillomavirus is?

For those who know, dig in on...

1. How is it spread/transmitted?

2. What health problems can it cause?

3. Have you heard about a vaccine for HPV? If so, what have you heard? Where did you hear about it?

How concerned are you about getting HPV?

**IF CONCERNED**: Why are you concerned?

(Dig in on specific concerns.)

**IF NOT CONCERNED**: Why do you feel like you aren’t concerned?

(Dig in on why not concerned.)

**ALL:** Can any of you tell me some ways to prevent HPV that you know of?

**ATTITUDES TOWARD VACCINES (start around 15-minute mark, 7-minutes):**

Thanks so much for sharing that! Now I’d love to talk about vaccines, generally.

How safe do you think vaccines are?

**FOR THOSE WHO THINK SAFE**: Why do you believe vaccines are safe?

(Dig in on vaccine safety beliefs and attitudes.)

**FOR THOSE WHO THINK UNSAFE**: Why do you believe vaccines are unsafe?

(Dig in on attitudes and beliefs on why vaccines are unsafe.)

In general, what are your views on the HPV vaccine?

(Dig in on how family or close friends share the same views.)

**FOR VACCINATED GROUP/PARTICIPANTS: (Trans/Non-Binary: For those who are vaccinated)…** Why did you get the HPV vaccine?

(For example was it a physician recommendation, their own motivation, knowing someone else who got it, etc.)

**FOR UNVACCINATED GROUP/PARTICIPANTS: (Trans/Non-Binary: For those who are not vaccinated)** Did you decide to not get the HPV vaccine or did it not occur to you? **(For those who decided):** Why did you decide to not get the HPV vaccine?

How effective do you think the HPV vaccine is at preventing serious diseases caused by HPV?

**FOR THOSE WHO BELIEVE EFFECTIVE**: Why do you believe it is effective?

(Dig in on effectiveness beliefs.)

**FOR THOSE WHO DO NOT THINK EFFECTIVE**: Why do you believe it is not effective?

(Dig in on beliefs on why not effective.)

**AWARENESS OF VACCINE ACCESS (7 minutes):**

Thanks for sharing your thoughts! Now, I’d love to hear from each of you about your perceptions of access to vaccines.

**FOR VACCINATED GROUP/PARTICIPANTS:** How did you go about getting the HPV vaccine? Also, where did you get the vaccines?

(Dig in for full process.)

**FOR VACCINATED GROUP/PARTICIPANTS:** Did you run into any issues when trying to get the HPV vaccine?

(Dig in for breakdown of experienced problems, and how overcome.

**FOR UNVACCINATED GROUP/PARTICIPANTS:** What problems do you think you might run into?

(Dig in for breakdown of anticipated problems. What solutions they have and what they would need.)

**HEALTH INFORMATION SEEKING (start around 30-minute mark -- 7 minutes):**

Thanks so much for those insights! Now I’d love to hear about how you get your health information, generally, and who you trust for health information.

**ALL**: I’d love to hear from everyone, what are your strategies for finding health information?

(Dig in for channels they prefer, why they prefer them, find out their process of finding health information. Do they go to different places for different kinds of information?)

**ALL**: What sources do you all feel are most trustworthy for health information?

(Dig in for *why* they find sources trustworthy, what makes them trustworthy to participants.)

**ROLE OF SOCIAL MEDIA (12 minutes):**

Now in thinking about your health information sources, I’d love to ask…

**ALL**: Have any of you ever used TikTok to search for health information or health advice?

(Dig in for *why* they chose to use TikTok for health advice. What did it offer that other sources don’t?)

**ALL**: Have any of you ever seen posts about the HPV vaccine or vaccines in general on TikTok?

(Dig in for more information on what they’ve seen, then dig on in if they think that information influenced their knowledge, attitudes, or behaviors?)

**ALL**: For those who haven’t, how would you react if you saw HPV vaccine content on TikTok?

**ALL**: If you have viewed HPV vaccine content, did you trust it? For those who haven’t, would you trust HPV vaccine content on TikTok?

(Dig in for more information on what made it trustworthy, who shared, etc?)

**ALL**: What would you like HPV vaccine content on TikTok to be about (such as, the risk of HPV, how to get the vaccine, who is eligible, etc.)?

**ALL**: What type of messenger would you trust for TikTok content about the HPV vaccine (such as, an influencer you like, a provider, youth, etc.)?

**ALL**: What would be a turn-off about HPV vaccine content on TikTok?

**ALL**: Would any of you be willing to ever post about or discuss the HPV vaccine on TikTok?

**CLOSING (start around 57-minute mark, 3 Minutes):**

**That’s it! Thank you so much for your time. Before we wrap up today…**

Do you have any additional feedback that you want to share with me today?

If YES – Discuss

If NO – Thank you.

Do you have any questions for me at this time?

If YES – Discuss

If NO – Thank them and end the group.

That is all we have for today. Thank you for your time. We appreciate you all for sharing your thoughts with us. Within the next two business days, you will receive a $100 gift card from Plaza Recruitment Inc. as a thank you for your participation today.
